# Friction of sea ice

**DOI:** 10.1098/rsta.2017.0336

**Published:** 2018-08-20

**Authors:** Erland M. Schulson

**Affiliations:** Thayer School of Engineering, Dartmouth College, Hanover, NH 03755, USA

**Keywords:** sea ice, static and kinetic friction, mechanisms and models

## Abstract

Static and kinetic friction play a fundamental role in sea-ice mechanics. The coefficient of static friction increases with hold time under normal load and is modelled in terms of creep and fracture of asperities in contact. The coefficient of kinetic friction exhibits velocity strengthening at lower speeds and velocity weakening at intermediate speeds. Strengthening is modelled in terms of asperity creep and hardness; weakening is modelled in terms of a progressive increase in the true area of contact wetted by meltwater produced through frictional heating. The concept is introduced of contact size distribution in which the smallest contacts melt first, leading to the onset of weakening; the largest melt last, leading to a third regime of kinetic friction and again to strengthening where hydrodynamics governs. Neither the static nor the kinetic coefficient is significantly affected by the presence of sea water. The paper closes with a few implications for sea-ice mechanics. The paper is based largely upon a critical review of the literature, but includes a more quantitative, physics-based analysis of velocity strengthening and a new analysis of velocity weakening that incorporates parameters that describe the (proposed) fractal character of the sliding interface.

This article is part of the theme issue ‘Modelling of sea-ice phenomena’.

## 1. Introduction

Friction and fracture play a fundamental role in ridging and rafting [1–3], in faulting [4,5] and in other modes of brittle failure of the winter sea-ice cover [6]. In each case, one asks: how large are the coefficients of static and kinetic friction, how much resistance does the ice offer to crack propagation, and what is the nature of these material properties? In this paper we address friction—its characteristics and underlying physical mechanisms. Given that relative movement within the winter sea-ice cover is generally characterized by velocities less than or equal to 0.1 m s^−1^ or less than or equal to about 10 km d^−1^ [7,8], we limit discussion to behaviour at lower speeds. We discuss both sea ice and freshwater ice, for, as will become apparent, there is little to distinguish the two variants.

**Figure 1.**
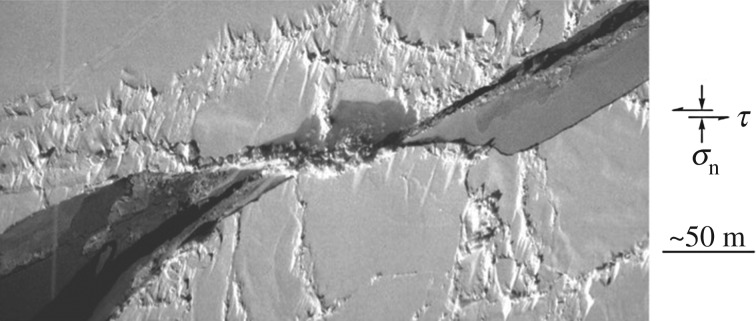
Aerial photograph of a region of the winter 2003 ice cover on the Beaufort Sea, centred approximately at 73° N, 148° W, showing a through-thickness crack across which left-lateral sliding occurred that led to the formation, from opposite ends, of two out-of-plane leads. The different grey levels within the leads are indicative of new ice of different thickness which, in turn, is indicative of intermittent sliding. The ‘parent’ crack is loaded globally by a normal stress *σ*_n_ and by a shear stress *τ*.

To set the scene, consider figure 1. The image shows a small region within the snow-covered winter sea-ice cover on the Beaufort Sea. Present is a deformation feature, horizontally oriented within the field of view and roughly 150 m in length, bordered by what appears to be a pile of fragmented ice/particles. From the two ends extend two leads, approximately 50 m wide, inclined to the horizontal by about 35° and covered with a thin layer of new ice. We interpret the feature to be a through-thickness crack, possibly of thermal origin, across which left-lateral sliding occurred intermittently (evident from the different thickness of the new ice within the leads) under the action of a wind-induced compressive stress that acted in a direction approximately parallel to the leads. The wind generated a shear stress, *τ*, that acted in the plane of the crack plus a normal stress, *σ*_n_, that tended to close the feature. Presumably, sliding (at a velocity not known) was initiated when the shear stress became large enough to overcome resistance offered by static friction and then continued while the shear stress remained large enough to overcome resistance offered by dynamic or kinetic friction. Sliding, we imagine, induced tensile stresses at opposite ends of the ‘parent’ crack: when large enough, tension was relieved through the initiation and stable growth of the out-of-plane extensions/leads, in the manner of wing-like cracks [9–11]. The fragments bordering the ‘parent’ crack are taken to be wear debris.

One can imagine other scenes where wind-driven stresses activate friction-based processes that culminate in both local and global fracture. In every case, as already noted, a question is: how large are the coefficients of static and kinetic friction and what is their nature?

## 2. Friction coefficient: a scale-independent property

Before proceeding, we note that many of the results to be described were obtained from measurements made in the laboratory. There, unlike the field, different factors can be explored systematically, thereby allowing the dominant ones to be identified. The question is whether the properties so measured and the interpretation that follows are reflective of the larger scale. One wonders because the brittle compressive strength of sea ice, for instance, to which friction contributes [4,12], is certainly different on the two scales and can be lower on the larger scale by up to a factor of 10^3^. That difference, however, is attributed not to friction, but to the presence of thermal cracks and other stress concentrators that are larger by up to a factor of approximately 10^6^.

**Figure 2.**
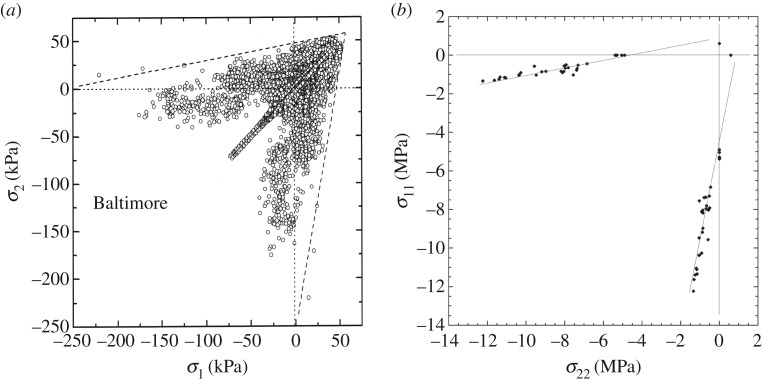
(*a*) Stress states within the winter sea-ice cover on the Arctic Ocean, measured *in situ* at the ‘Baltimore’ site by Richter-Menge *et al*. [14] and plotted in principal stress space; (*b*) low-confinement, Coulombic branch of the brittle compressive failure envelope (plotted in principal stress space) of columnar-grained S2 first-year sea ice harvested from the winter 2003 ice cover on the Arctic Ocean and then loaded biaxially across the columns to terminal failure in the laboratory at −10°C 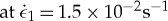, obtained by Schulson *et al*. [12]. Note that the slope of the field-derived and laboratory-derived envelopes is the same (adapted from Weiss *et al*. [13]).

**Figure 3.**
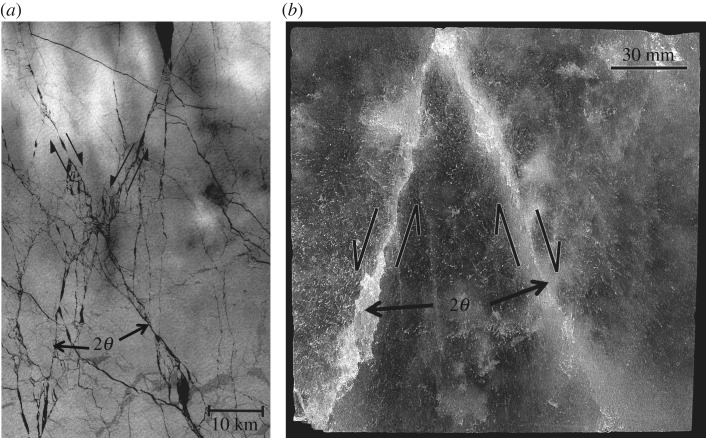
(*a*) Landsat-7 image obtained on day 84 of 2000 (24 March 2000) showing a set of intersecting linear kinematic features/Coulombic shear faults (or conjugate faults) within the winter sea-ice cover on the Arctic Ocean, centred approximately at 80° N, 135° W; north is approximately vertical. Sliding occurred in a right-lateral and a left-lateral manner across the two faults, evident from the rhomboidal shape of the openings along the features. The image was obtained by the Canadian RADARSAT satellite and was provided by R. Kwok of Jet Propulsion Laboratory. (*b*) Photograph showing a set of intersecting/conjugate Coulombic shear faults within ice harvested from the winter sea-ice cover and then compressed to terminal failure in the laboratory, as described in the caption to figure 2*b*. The long axes of the columnar-shaped grains are normal to the image and the greater (lesser) compressive stress is oriented vertically (horizontally). Note that the angle of intersection, 2*θ*, is similar in the two images. (From Fortt [15].)

About friction *per se*, two observations—albeit indirect—are relevant. The first is the slope of the low-confinement branch of the brittle compressive failure envelope, d*σ*_1_/d*σ*_3_ (where *σ*_1_ and *σ*_3_ denote the most compressive and least compressive principal stresses, respectively). Whether derived from measurements made in the laboratory on ice harvested from the winter sea-ice cover or from measurements made in the field of the stresses within the winter cover, the slope is essentially the same [13] (figure 2). The slope of the envelope is governed solely by the coefficient of kinetic friction, *μ*_k_, through the relationship [12] 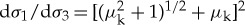. (Note from figure 2 that the field stresses are lower by a factor of approx. 10^3^, as mentioned above.) Also, figure 2 plots σ_2_ and σ_22_ instead of σ_3_, because S2 ice deforms in plane-strain when loaded across the columns. The other relevant observation is the included angle of intersection, 2*θ*, of conjugate Coulombic shear faults. Again, whether measured from deformation features generated in the laboratory or from satellite images of intersecting sets of linear kinematic features/faults, 2*θ* is almost the same (figure 3). This characteristic, too, is governed solely by the coefficient of kinetic friction, through the relationship [16] 2*θ *= arctan(1/*µ*_k_. Proof of left-lateral and right-lateral slips along intersecting faults is the sets of rhomboidal-shaped openings that punctuate them (see figs 2 and 5 of [4]).

We proceed, therefore, with the view that friction in the laboratory, if not exactly the same as in the field, is close enough to offer a reasonable depiction of the larger scene, at least in terms of the physical factors and fundamental mechanisms that govern behaviour.

## 3. Static friction: characteristics

The coefficient of static friction, *μ*_s_, increases with time under load. This is evident from experiments performed in three different arenas: in the laboratory [17–19], in an ice tank [17] and in the field [20,21].

**Figure 4.**
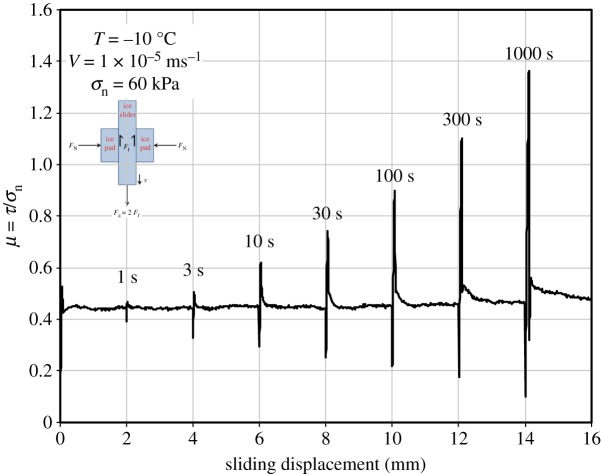
Results from slide-hold-slide tests at −10°C on S2 columnar-grained, first-year sea ice harvested from the winter 2009 ice cover on the Beaufort Sea, centred at approximately 71° N, 156° W. The velocity of sliding across the columns (in a direction perpendicular to the long axes of the grains) was 1 × 10^−5^ m s^−1^; the normal stress was *σ*_n_ = 60 kPa. The hold time ranged from 1 s to 1000 s. The graph plots the ratio of the shear stress to the normal stress, *μ* = *τ*/*σ*_n_, versus displacement. Note that the stress ratio at the onset of sliding after holding, taken as the coefficient of static friction, increases with holding time and that the ratio during sliding, taken as the coefficient of kinetic friction, remains relatively constant. (From Schulson and Fortt [19]). (Online version in colour.)

**Figure 5.**
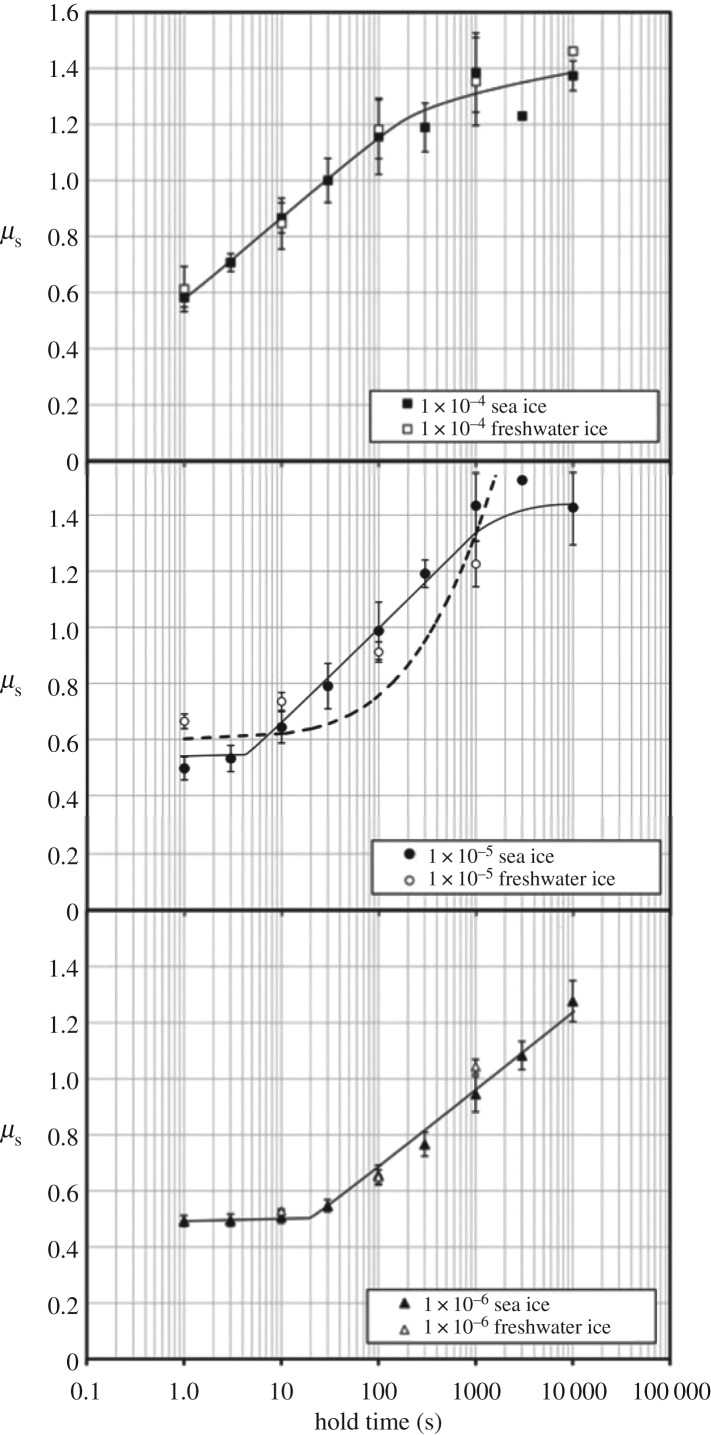
Coefficient of static friction, *μ*_s_, versus hold time (derived from the slide-hold-slide measurements of the kind shown in figure 4) for both the first-year sea ice described in the caption to figure 4 and granular, freshwater ice, at −10°C sliding velocities of 10^−6^, 10^−5^ and 10^−4^ m s^−1^ . The dashed curve in the central panel was calculated from equation (4.5). Note that following a threshold period static friction increases linearly with the logarithm of hold time, up to some upper time limit, and that the threshold period increases with decreasing sliding velocity. Note also that within the scatter in the measurements, sea ice could not be distinguished from freshwater ice. (From Schulson and Fortt [19].)

To illustrate this point, figures 4 and 5 show results that were obtained from slide–hold–slide (SHS) experiments performed in the laboratory on first-year, S2 columnar-grained sea ice (loaded across the columns, 6.1 ± 2.3 mm column diameter, density 918 ± 4 kg m^−3^ at −10°C, salinity 4–5 p.p.t., surface roughness *R*_a _= 0.76 ± 0.51 × 10^−6^ m) and, for comparison, results from freshwater granular ice (1.5 ± 0.5 mm grain size, density 910 ± 4 kg m^−3^, *R*_a _= 0.43 ± 0.24 × 10^−6^ m). The sea ice had been harvested from the 2009 winter ice cover on the Beaufort Sea, at 71° N, 156° W and then, to simulate nature, was slid in such a manner that the long/vertical faces of the columnar grains rubbed against each other in a direction perpendicular to the long axes of the grains. The experiments were performed at −10°C using a using a double-shear device attached to a servohydraulic actuator. For both materials, the interface was held under a low normal stress of *σ*_n_ = 60 kPa, reflective of the natural loading of the arctic sea-ice cover [14]. Hold time ranged from a few seconds up to approximately 3 h, following intermittent sliding at velocity of either *V*_s _= 10^−6^, 10^−5^ or 10^−4^ m s^−1^. The coefficient of static friction, just before sliding was reinitiated, was defined as the ratio of the friction shear force *F*_f_ divided by the normal load *F*_n_ (where, owing to double shear, *F*_f_ is one-half the applied force *F*_a_ to reinitiate sliding); i.e. 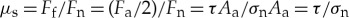 where, again, *τ* and *σ*_n_ denote the global shear stress and global normal stress, respectively, acting on the interface and *A*_a_ denotes the apparent contact area. The results from these and other SHS tests revealed the following characteristics:

(i) An increase in the coefficient of static friction, *Δμ*_s_, termed *static strengthening*, is detected once hold time exceeds a threshold period, *t*_t_; the threshold decreases with increasing velocity, from (under the conditions of the laboratory tests) *t*_t_ = 30 s at *V*_s _= 10^−6^ m s^−1^ to *t*_t _= 3 s at *V*_s _= 10^−5^ m s^−1^ to *t*_t _∼ 3 s at *V*_s _= 10^−4^ m s^−1^.
(ii) Static strengthening increases with hold time, *t*_h_, scaling logarithmically as Δ*μ*_s_ = *μ*_s_ −*μ*_os_ = *β*log_10_*t*_h_ for *t*_t_ < *t*_h_ < *t*_u_ where *μ*_os_ denotes a reference coefficient, *t*_u_ denotes the upper limit on time for logarithmic dependence at which point the coefficient appears to level off; *β* = 0.30 ± 0.03 under the laboratory test conditions. Alternatively, the strengthening may be described by the power-law Δ*μ*_s_ = *At^m^*, where *m* = 0.5 ± 0.1 for *t*_t_ < *t*_h_ < *t*_u_ and *A* is a constant.
(iii) The shear stress required to continue sliding is little affected by holding, implying that the coefficient of kinetic friction (more below) is independent of holding under load.
(iv) Within scatter in the data, the coefficient of static friction of sea ice is essentially indistinguishable from that of freshwater ice.
(v) The presence of sea water at the interface (at least of metre-sized specimens) has little detectable effect on the coefficient of static friction [21].

On interfacial rubble (similar to the kind depicted in figure 1), Scourfield *et al*. [22] performed a series of double-shear experiments in which fragments of ice were placed between opposing faces of sea ice. The tests were performed in the Barents Sea during March 2014 when the air temperature ranged between −7°C and −10°C. They pulled a metre-sized block of floating sea ice through a channel of open water. Hold time varied from 1 s to 16 h. Again, static strengthening was detected. Logarithmic behaviour was obeyed during the earlier stages, but the sensitivity to time increased after approximately 3 h, owing perhaps, as Scourfield *et al*. suggest, to consolidation. Unfortunately, neither normal load nor measurements for rubble-free behaviour were reported. Hence the question about rubble and its role in the coefficient of static friction remains unanswered.

In exhibiting static strengthening, ice on ice is not unique. Similar behaviour has been detected when ice is pressed against steel, against PMMA and against rubber at temperatures from −13.5°C to −3.5°C [23,24]. Static strengthening is seen also in non-icy materials when pressed together, including a variety of rock and minerals [25,26], metals [27] and glassy polymers [28]. In those material systems, too, the effect is characterized by logarithmic dependence on hold time, although the strengthening coefficients, *β*, are lower by about an order of magnitude. The lower values probably reflect the lower homologous temperatures at which the experiments were performed. Indeed, the strengthening coefficient of cold ice is lower than that of warm ice [18], falling to *β* = 0.10 at −75°C (*T*_h_ = 0.72) and to *β* ∼ 0 at both −100°C (*T*_h_ = 0.63) and −175°C (*T*_h_ = 0.36).

## 4. Static friction: physical mechanisms

**Figure 6.**
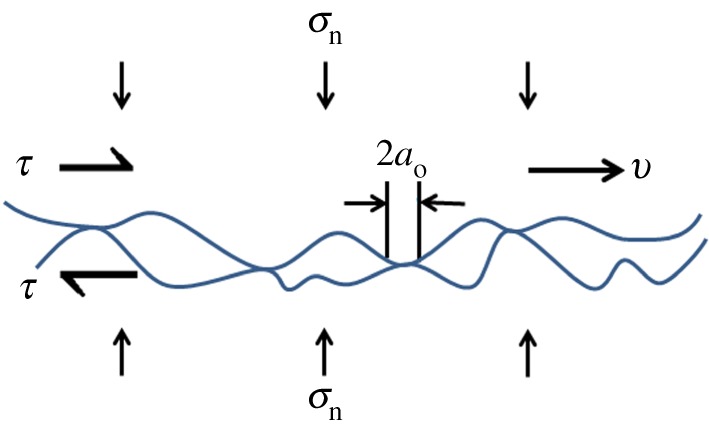
Schematic sketch of asperities of initial diameter 2*a*_o_ in contact across an interface subjected to a global normal stress *σ*_n_ and to a global shear stress *τ* (as in figure 1). The opposing faces are displaced at a constant velocity *v* in the horizontal direction. (Online version in colour.)

Bowden & Taylor [29,30] proposed that friction originates from the interaction of micro-asperities that protrude from opposing surfaces in contact, as sketched in figure 6. Accordingly, static strengthening of warm ice can be understood in terms of creep and fracture of asperities that support the load. In essence, the greater the hold time and the warmer the ice, the greater is the degree to which asperities shorten through creep deformation. Correspondingly, owing to the conservation of volume, the greater the shortening the greater is the increase in the real contact area. Assuming that cohesion occurs rapidly, the greater the contact area, the greater is the shear stress required to rupture the asperities and hence the greater is the coefficient of static friction.

### (a) Creep deformation

To quantify this explanation, Schulson & Fortt [19] invoked power-law creep. They suggested that asperities in contact fail in tension and not in shear per se. A multiaxial stress state is induced by stresses that are generated through the combined action of shear stress and normal stress acting locally. Accordingly, for asperities of height *l* the rate of creep is given by 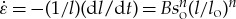, where *s*_o_ denotes effective stress at time zero. Upon introducing through conservation of volume *l* = *l*_o_(*a*_o_/*a*)^2^ and then integrating, the ratio of final to initial contact area, (*a*/*a*_o_)^2^, for hold time *t*_h_, is given by the following relationship [19]:
4.1
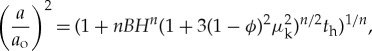

where *n* denotes the stress exponent in the power-law relationship for creep rate (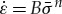
where *B* is a temperature-dependent materials parameter, 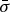 is the local effective stress acting on the asperities given by 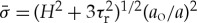 where *H* denotes hardness which is equated to the normal compressive stress supported by each asperity, *τ*_r_ is the residual shear stress *τ*_r_ = *μ*_k_*H* that acts locally on the asperities during holding and *μ*_k_ is the coefficient of kinetic friction) and *ϕ* is the factor that accounts for stress relaxation during holding, 0 ≤ *ϕ* ≤ 1.

### (b) The coefficient of static friction

The coefficient of static friction may then be given by the relationship
4.2
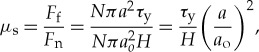

where *N* denotes the number of contacts; *τ*_y_ is the magnitude of the local shear stress at the point of rupture and is given by
4.3
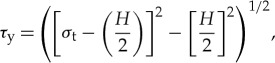

where *σ*_t_ denotes the tensile strength of the asperity. Thus, from equations (4.1)–(4.3), the coefficient of static friction versus hold time may be given by the relationship
4.4
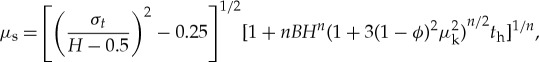

(Note that in equation (4.4) *H* has a negative value in the first term, to reflect the stress tensor, but a positive value in the second term to reflect creep stress.) Upon inserting appropriate parametric values for ice at −10°C, albeit for bulk material and not specifically for asperities (from Barnes *et al*. [31]: *n *= 3, *B *= 4.3 × 10^−7^ MPa^−3^ s^−1^, and |*H*| = 20 MPa; *σ*_t_ = 6 MPa [32]; *μ*_k_ = 0.5, more below), and upon assuming *ϕ* = 0.5, equation (4.4) reduces to
4.5
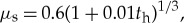

for hold time in seconds. The parameter *ϕ* is the only ‘free’ parameter in the model and is a relatively unimportant one, for if reduced to zero (no relaxation) or increased to unity (complete relaxation) the computed coefficient differs only by about 10%.

Equation (4.5) is shown as the dashed curve in figure 5, for a sliding velocity of *V*_s_ = 10^−5^ m s^−1^. For hold time *t*_h_ ≤ 1000 s, the model captures the experimental measurements reasonably well. It is consistent, also, with the observation that sea water at the interface has either little or no detectable effect, for there is no evidence that ice creeps more rapidly when wet. (In fact, creeping asperities within submerged ice may not be wet at all, for water present at the interface may have been squeezed out as asperities come in contact.) However, the model overestimates (underestimates) by about 20–40% the lowest (highest) velocity SHS tests (at 10^−6^ and 10^−4^ s^−1^), discrepancies that could be attributed to the *n*-value and to whether a single value applies over the entire period of holding. Also, the model does not include the trend towards saturation. Missing, too, is log-time dependence. (That feature, as noted by Brechet & Estrin [33], requires creep to be described by an exponential function and not by a power-law.) Despite these shortcomings, the creep-based model of static friction appears to work sufficiently well to justify further development along these lines.

Sea ice creeps more rapidly than freshwater ice, by about an order of magnitude [34,35]. Why, then, are the coefficients of the two variants essentially indistinguishable? The reason, we suggest, is that asperities in sea ice, owing to their small size, do not contain creep-enhancing brine pockets and hence may be regarded as protuberances of freshwater ice.

## 5. Static healing

An implication of the above analysis is that strength can be restored to damaged sea ice upon holding under load. To explore that point, experiments were performed [36] on a laboratory-grown analogue of first-year sea ice; namely, on columnar-grained S2 saline ice of 4–5 ppt salinity into which Coulombic shear faults were introduced. The faults were healed by confining the ice across the columns under a balanced, biaxial compressive stress *σ*_1_ = *σ*_2_ =*σ*_h_ of 60–750 kPa and then holding for a period from *t*_h_ = 3 s to 18 h at temperatures from −30°C to −3°C. Subsequently, the unconfined, brittle compressive strength of the faulted and partially healed material was measured.

The restored strength of the damaged ice, *σ*_r_, scales linearly with confining stress and increases with both increasing time and temperature [36]. When normalized with respect to the strength of fault-free material, σ*, the restored strength can be described by the relationship
5.1
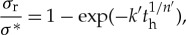

where *k′* and *n′* are constants for a given set of conditions and *t*_h_ again denotes time under load. Modelling, again in terms of creep-driven increase in the real area of contact, leads to the short-time limit [36]
5.2
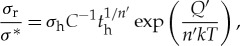

where *C* is a materials constant, *Q′* is an apparent activation energy, *k* is Boltzmann's constant and *T* is the absolute temperature. Strengthening/healing kinetics yielded parametric values *C* = 0.027 MPa s^1/*n*^′, *n′* = 3.4 and *Q′* = 50 ± 6 kJ mol^−1^. The values derived for *n′* and *Q′* are similar to those derived by Barnes *et al*. [31] from creep-based hardness kinetics, lending credibility to the interpretation.

Interestingly, healing kinetics exhibit little dependence upon interface roughness, at least over the relatively small range 0.06≤ *R*_a_ ≤ 0.26 mm. This suggests that, like the case for ductile metals [37–39], there are smaller asperities superimposed upon larger asperities at all scales.

The restoration of strength implies that molecular bonds form at points of contact. In turn, this implies that the cohesive strength in Coulomb's Law (§6b) is increased upon healing, but that the coefficient of kinetic friction is not affected. This is consistent with the observation (figure 4) that the shear stress required to continue sliding after holding is essentially independent of hold time.

## 6. Kinetic friction: characteristics

Once static friction has been overcome, sliding ensues. In the discussion below, velocity is taken to be constant at the macroscopic level. The discussion is limited to lower-speed sliding. Higher-speed sliding of the kind related to winter sports has been reviewed by Kietzig *et al*. [40].

### (a) Stick-slip sliding

Within the low-speed regime (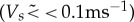 ), sliding generally occurs in a stick-slip manner, marked by rapid oscillations in shear force of 10% or more. Stick slip appears to be ubiquitous, characterizing ice in a variety of forms within a number of arenas—including the ice cover on the Arctic Ocean [1,41,42]; metre-sized blocks harvested from the winter ice cover and pulled over the cover [20,21]; the thin cover of an ice tank [17,43]; and sub-metre-sized specimens of both saltwater and freshwater ice sliding in the laboratory across interfaces both smooth (*R*_a_ ∼ 1 µm) and rough (*R*_a_* *∼ 1 mm) [19,44–46]. We expect that stick slip is accompanied by acoustical emissions, although that feature remains to be explored.

Stick slip is a dynamic instability. It is characteristic not only of ice, but of the compliance of the loading system. Its nature, however, has not been studied systematically and hence meaningful discussion must await future work. Stick slip implies that, while the overall velocity imposed by the loading system (e.g. the actuator of a servohydraulic system used in the laboratory) may be constant, the relative displacement across the interface varies somewhat as elastic energy is stored and then released.

Although stick slip imposes temporally variable shear stresses, in the description below we use a temporal average over many cycles to quantify this parameter, in keeping with common practice.

### (b) The friction law

To determine the kinetic friction law, measurements have been made under a variety of conditions of the average global shear stress, *τ*_s_, versus the global normal stress, *σ*_n_. The conditions include both sub-metre and metre-sized specimens of first-year sea ice and freshwater ice, both wet and dry, sliding in the laboratory and in the field at temperatures around −10°C and −40°C at velocities of *V*_s_ = 8 × 10^−4^ m s^−1^ and 8 × 10^−2^ m s^−1^ [21,47] as well as cold, freshwater ice sliding in the laboratory at temperatures as low as −175°C at velocities from *V*_s_ = 5 × 10^−8^ to 1 × 10^−3^ m s^−1^ [18]. Under every condition explored, and within scatter in the data, the shear stress increased linearly with increasing normal stress over the range examined, from *σ*_n_ = 2 kPa to 2 MPa (see fig. 8 of ref. [47], fig. 4 of ref. [48], fig. 9 of ref. [21] and fig. 5 of ref. [18]). This implies that ice obeys Coulomb's friction law:
6.1
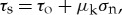

where *μ*_k_ denotes the coefficient of kinetic friction and *τ_o_* is the cohesive strength. Cohesion does not contribute significantly, except during sliding at the lowest velocities at the highest temperatures [18].

There are instances in which non-linear behaviour has been reported, where 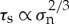 [44,49,50]. In those cases, the normal stress was higher than noted above and was in the range of 5–250 MPa. The departure from linearity may reflect the activation of additional energy dissipative mechanisms.

### (c) Physical factors

**Figure 7.**
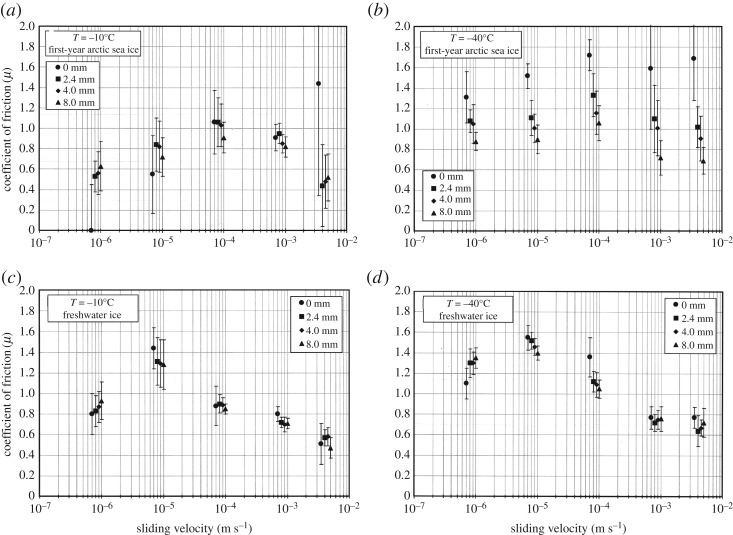
Coefficient of kinetic friction versus sliding velocity for first-year arctic sea ice (harvested as described in the caption to figure 4) and S2 columnar-grained freshwater ice at −10°C and −40°C, measured after sliding distances of 0, 2.4, 4.0 and 8.0 mm across Coulombic shear faults of roughness *R*_a _∼ 1 mm. The points are displaced for clarity. Note the increase and then decrease in the coefficient with increasing velocity. (From Fortt and Schulson [47]).

The primary factor that affects the coefficient of kinetic friction under terrestrial conditions is sliding velocity; its effects are described below. On other factors, temperature is important: it leads to an increase of about 20 to 40% upon dropping from −10 to −40°C [18,47] and to a decrease of about 10–20% upon increasing to −3°C [48]. Also, roughness has an effect [18]: over the range examined (*R*_a_ = 0.4 − 1200 µm) and under the conditions explored (−10°C, *V*_s_ = 1 × 10^−4^ m s^−1^), the coefficient scales as 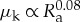, leading to about a factor of two difference between rough (*R*_a_ ∼ 1 mm) Coulombic faults and smooth/milled (*R*_a_ ∼ 1 µm) surfaces (more below). Furthermore, a cover of snow increases *μ*_k_ by about a factor of 1.3, at least when sliding over warm arctic sea ice at about *V*_s_ = 1.5 × 10^−2^ m s^−1^ [20], reminiscent of the effect of snow on waxed hickory sliding at a velocity of 0.1 m s^−1^ [51]. On the other hand, grain size and grain shape [18,45,52] have no detectable effect. Nor does the presence of water external to the interface [21], reminiscent of the absence of an effect of sea water in the ice/steel and ice/concrete material systems [53]. Similarly, sliding distance, at least up to the limit of 8 mm examined to date, exhibits little or no detectable effect [47] (figure 7). Furthermore, salinity at the level of ≈5 ppt found in sea ice exhibits little detectable effect, reminiscent of static friction.

### (d) The effect of velocity

Low-speed sliding of warm ice (homologous temperature of around *T*_h_ ≥ 0.8) exhibits two kinds of behaviour. At the lowest speeds, i.e. at velocities lower than about *V*_s_ = 10^−5^ to 10^−4^m s^−1^, depending upon temperature, the coefficient of kinetic friction increases with increasing velocity. This behaviour we term *velocity strengthening*. At higher speeds, the coefficient decreases with increasing velocity, termed *velocity weakening*. Figure 7 shows examples of this behaviour for both first-year sea ice and freshwater ice of similar microstructure sliding across Coulombic shear faults over relatively rough interfaces ( *R*_a _∼ 1 mm) at −40°C and at −10°C. (Early work at low speed/high temperature did not report velocity strengthening, presumably because the sensitivity to velocity is lower within the strengthening regime and because the coefficient was derived not from the slope of a *τ*_s_ − *σ*_n_ curve, but from the ratio *μ*_k_ = *τ*_s_/*σ*_n_; the ratio overestimates the coefficient when cohesion becomes significant.)

**Figure 8.**
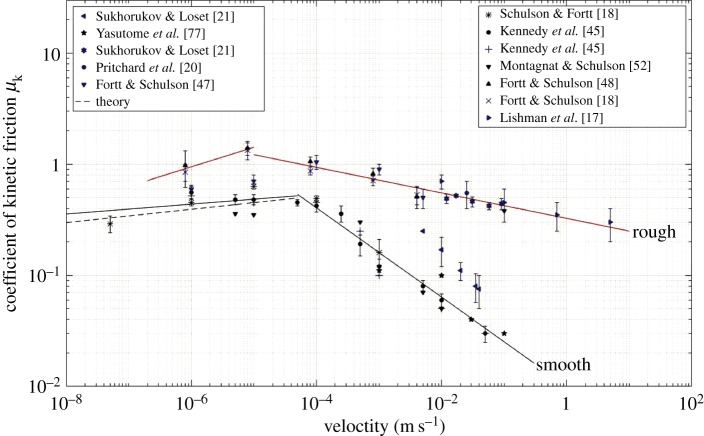
Coefficient of kinetic friction at −10°C (or thereabouts) versus sliding velocity for both sea ice (blue points) and freshwater ice (black points). The solid curves describe the data obtained from smoother (*R*_a _∼ 1 µm) surfaces (black line) and rougher (*R*_a _∼ 1 mm) surfaces (red line). The dashed curve over the range of lower velocities was calculated from equation (7.2).

Figure 8 summarizes many of the measurements to date at −10°C (or thereabouts) and offers a more complete picture. Included are coefficients measured from both smooth (*R*_a _∼ 1 µm) and rough (*R*_a _∼ 1 mm) surfaces on both saltwater and freshwater ice plus data obtained in the field and in the laboratory. As well as the trends of strengthening and weakening noted above, the data indicate that the rather moderate 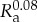 -proportionality noted above appears to be limited to the strengthening regime; within the weakening regime, roughness appears to have a greater effect, where the coefficient scales as approximately 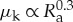. The reason for the increased sensitivity is not known, but may be related to sliding-induced fragmentation that sets in at the higher speeds (more below) and possibly to larger fragments from rougher surfaces. Figure 8 supports the point that from the practical perspective the kinetic coefficients of sea ice and freshwater ice, like the static coefficients, are practically indistinguishable.

In exhibiting dual behaviour, again ice on ice is not unique. The material systems ice/glass and ice/granite [31] behave in a similar manner, as do the non-icy systems of rock on rock [54–60]. Indeed, the parallels with rock are quite marked, even at the quantitative level, as evident upon comparing figure 8 with fig. 3 of DiToro *et al*. [56]. In addition, from tests at higher velocities only (*V*_s _> 10^−4^ m s^−1^), ice/steel, ice/concrete, ice/rubber and ice/nylon [40,53,61] all show velocity weakening.

### (e) Sliding-induced damage

To the unaided ear, sliding within the upper range of the velocity weakening regime is rather noisy [48]. It is accompanied by the production of gouge-like debris/granulated ice [45] and by the formation of short cracks that extend to a depth of approximately 0.5 mm [45,52]. In other words, fracture enters the picture over the lower part of the weakening regime.

## 7. Kinetic friction: physical mechanisms

### (a) Overview

**Figure 9.**
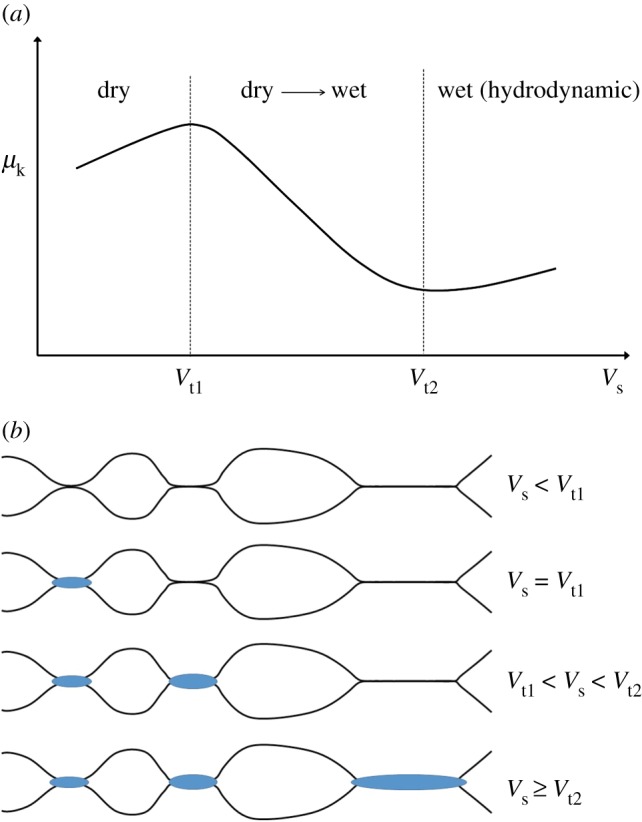
Schematic sketches illustrating (*a*) the three regimes of the kinetic friction of ice, dry, dry->wet, wet, and (*b*) the proposed increasing amount of meltwater (blue) with increasing velocity on contacts of increasing size.

In exhibiting two regimes of behaviour—indeed three regimes when high-speed sliding is included [40]—kinetic friction is seen to be more complicated than static friction. Again, the interaction of asperities as envisaged by Bowden & Taylor [29,30] is fundamental to the process, but in a manner that differs with velocity. At the lowest speeds, the time for one asperity to pass over another is sufficiently long to allow the frictional heat generated during passage to be conducted away from the interface. Contacts remain solid and dry sliding ensues. At a critical velocity, *V*_t1_, affected possibly by the liquid-like layer that forms on the surface of ice near its melting point [62], insufficient time is available to conduct away all frictional heat. At that point and beyond, contacts begin to melt and resistance to sliding decreases. At a second critical velocity, *V*_t2_, greater than the first, a layer of water, we think, covers all original contacts. At that point, hydrodynamics governs [63–65] and sliding resistance once again increases with velocity [40,63,64]. Figure 9 sketches this scenario. In the discussion below, the rate of displacement across the interface on both local and global scales is taken not to vary for a given imposed velocity, despite the stick-slip character of sliding.

### (b) Dry sliding: velocity strengthening

#### (i) A thermodynamic model

Makkonen [66] and Makkonen & Tikanmaki [67] modelled dry sliding in terms of thermodynamics. The idea is that resistance is controlled by surface energy, specifically by the energy of surface steps or ledges that are created at the nanoscale. Accordingly, the coefficient of friction is expressed as *μ*_k_ = *γ*/*Hλ* where *γ* denotes surface energy, *H*, the hardness and *λ*, a nanoscale contact length. Although data for *λ* are not available, it was assumed that this parameter is equal to the size of the smallest cluster of H_2_O molecules that is stable in the solid state. Based upon calculations by Pradzynski *et al*. [68], this size is of the order of *λ* = 2 nm. Taking this value and taking the values *γ *= 75 mJ m^−2^ and *H *= 60 MPa (where 60 MPa corresponds to hardness [31] for an indentation time of approx. 10^−4^ s (which is about the amount of contact time *t* = *λ*/*V*_s_ to be expected for sliding at *V*_s_ ∼ 10^−5^ m s^−1^ across an asperity approx. 2 nm in diameter)), the thermodynamic model dictates *μ*_k_ = 0.6 for dry sliding at −10°C. This value agrees reasonably well with measurement (figure 8). The problem is that the thermodynamic model does not account for velocity strengthening. The only parameter that exhibits rate dependence is hardness and that property is expected to increase with decreasing time or increasing velocity [31], assuming that nano- and micro-scale features mimic ice in bulk form. An increase of two orders of magnitude in sliding velocity, for instance, is expected to lead to an increase of about a factor of two or more in hardness (more below) and, on the above model, to a reduction in the friction coefficient by the same factor. The thermodynamic model, in other words, dictates an effect of velocity on dry sliding of opposite sign to the one observed.

#### (ii) A creep-based model

A different explanation-cum-model is based upon creep of asperities and of underlying material [31,45,69]. Again from equation (4.2), *μ*_k_ = *τ*_y_/*H*. At low rates of loading both the shear strength, *τ*_c_, and the hardness of ice increase with increasing strain rate, 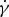, or with decreasing loading time, *t*. The rate-dependencies are expressed as [31] 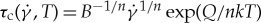 and 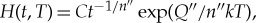 where, as above, *B, C, n* and *n′′* are material constants, and *Q* and *Q′′* are activation energies for creep and hardness, respectively. Upon substituting 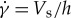, where *h* is the thickness of the near-surface inelastic zone, and *t* = 2*a*/*V*_s_, where again *2a* denotes contact diameter (assumed not to increase significantly at the low velocities of dry sliding), it follows that
7.1
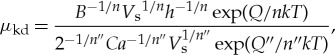

where the subscript ‘d’ denotes dry sliding.

To evaluate the model requires asperity size, *2a*. A value can be obtained from static strengthening experiments (§3), as follows [18]: given that the total time for asperity creep is the sum of the time for asperities to slip past each other plus the time of holding, the effect of holding per se is not detected until the hold time exceeds the slip time. In other words, the threshold period, *t*_t_, described in §3a that marks the beginning of detectable static strengthening also helps to define asperity size. The relationship is 2*a* = *t*_t_*V*_s_ = 30 × 10^−6^ = 3 × 10^−5^ = 0.3 × 10^−4^ = 30 µm. (Interestingly, although contacts probably range in size (more below), an average diameter of 30 µm is within the range 1–100 µm found for ductile metals [37,70] and for rocks and minerals [71], lending credibility to the size derived for ice).

Thus, setting 2*a* = 30 × 10^−6^ m and using creep data of Treverrow *et al*. [72] and hardness data of Barnes *et al*. [31], taking *Q* = 0.81 eV and *Q′′* = 0.75 eV [31], setting *h* = 100 µm [31] and using the value *n *= 3.5 [72] for tertiary creep (on the basis that asperity strain *γ* = 2*a*/*h* approx. 0.3 is well beyond the transition to tertiary creep) and taking the value *n′′* = 4.4 [31], we derive for ice at −10°C the values *B* = 2.5 × 10^15^ Mpa^−3.5^ s^−1^ and *C* = 0.027 MPa s^1/*n*^. Equation (7.1) then gives for the coefficient of dry kinetic friction at −10°C the relationship
7.2
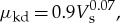

for velocity in units of m s^−1^.

Equation (7.2) is shown as the dashed line on figure 8. Barring roughness, which remains to be incorporated, the model captures the dry-sliding measurements quite well.

The fact that the kinetic friction of sea ice and freshwater ice is practically indistinguishable, even though bulk sea ice creeps more rapidly, is again taken to mean that asperities and the underlying deformation zone are too small to contain creep-enhancing pockets of brine.

### (c) Dry–wet sliding: velocity weakening

As already mentioned, fundamental to velocity weakening is frictional heating and the creation of meltwater. Yet, direct observations of water at the sliding interface are lacking. There is, however, indirect evidence, deduced from Coulombic shear faults. When introduced into relatively warm (at −10°C) specimens of both sea ice and freshwater ice through proportional, biaxial loading, the faults did not come apart [47]. They had slid a few millimetres in about one second, i.e. at a velocity of approximately 10^−3^–10^−2^ m s^−1^ . This speed is within the weakening regime. Solid-state healing of the kind discussed in §5 could not have been at play, for the contact time was too short. Instead, cohesion probably originated in the freezing of frictional-produced meltwater upon uploading the ice.

#### (i) Current models

Current models that incorporate meltwater are based on the concept of thermal balance at contacts and the conduction of frictional heat into the ice [63,64,66,67]. The models incorporate the view that some of the frictional energy is expended through the creation of a layer of meltwater whose thickness increases with increasing velocity. Friction is then taken to be governed by conduction of frictional heat into adjacent material. That view leads to expressions of the form 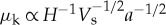 where the parameters have the same meaning as above. In dictating 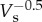-dependence, the models reflect the observation, from figure 8, that 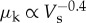, at least for sliding on smoother surfaces**.** The issue, however, concerns quantitative agreement. To conform to measurement, the contact size was set [67] to 2*a* = 1 mm without linking this size to any underlying physics.

A better model, we think, incorporates asperities of a range of sizes. Hatton *et al*. [73] envisaged asperities of different heights and curvatures, and then developed a model that, while retaining the role of meltwater as a lubricant, incorporates a principle of maximum displacement of deformation normal to the asperities and of minimum stress for shear failure. The model captures velocity weakening as well as velocity strengthening. However, it leads to two dictates that conflict with observation: that the coefficient of kinetic friction begins to decrease at *V*_s _∼ 10^−2 ^m s^−1^ at −5°C, compared with the observed onset at velocities two to three orders of magnitude lower, albeit at −10°C; and, that the value of the coefficient approaches zero at velocities lower than approximately 4 × 10^−3^ m s^−1^ compared with measured values at that speed that range from *µ*_k _∼ 0.1 (smooth interface) to 0.6 (rough interface).

#### (ii) The critical velocity

Rather than a range of asperity heights, we propose that velocity weakening is related to a variation in contact area. The smallest contacts, we imagine, melt when *V*_s_ = *V*_t1_, the largest when *V*_s_ = *V*_t2_. To remain wet, the time for asperities to slide over each other, *t*_s_, must be shorter than the time to transfer the latent heat of fusion via conduction into the adjacent ice, *t*_c_. The passage time is given by *t*_s_ = 2*a*/*V*_s_. The conduction time from a one-dimensional model of heat transfer may be estimated from the relationship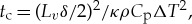 where *L*_v_ denotes the latent heat of fusion per unit volume, *δ* the thickness of the meltwater layer, *κ* the thermal conductivity of ice, *ρ* the density of ice, *C*_p_ the specific heat of ice and Δ*T* the difference in temperature between the melted contact (0°C) and the adjacent ice; the factor of 2 takes into account heat transfer into both sides of the contact. Upon setting *t*_s_ = *t*_c_, it follows that the velocity *V*_sc_ that marks the contribution to weakening from meltwater that forms on contacts of diameter *2a* scales as *V*_sc_ ∝ *a*Δ*T*^2^ and is given by the relationship
7.3
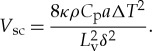

A range of contact sizes leads to a range of velocities over which the areal fraction of wet contacts changes. (We did not invoke a range of sizes in discussing both static friction and dry kinetic friction, because in modelling creep, we assumed that an average size captures the physics of the process well enough.)

Incidentally, although cold ice is beyond the scope of this discussion, we note that velocity weakening is not detected in freshwater ice sliding against itself at velocities up to *V*_s _= 10^−3 ^m s^−1^ at temperatures of −100°C and lower [18]. The absence of weakening within cold ice sliding at the speeds noted is consistent with the model, for equation (7.3) dictates that to a first approximation weakening of the colder ice would not be expected until the velocity exceeded the critical velocity of the warmer ice by a factor of (Δ*T*_cold_/Δ*T*_warm_)^2^ = (100/10)^2^ = 100 or greater.

#### (iii) The coefficient of kinetic friction: fractal model

Returning to warm ice and to the presence of meltwater, the coefficient of kinetic friction may be described by a rule of mixtures
7.4
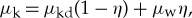

where *μ*_kd_ is the kinetic coefficient for dry contacts, given by equation (7.1), *μ*_w_ is the kinetic coefficient of water on ice and *η* denotes the fraction of the real area of contact covered by a layer of meltwater.

To quantify *η*, we imagine that the sliding interface has fractal character. Accordingly, and in keeping with the description of natural and prepared surfaces in other materials (e.g. see [74] and references therein), the distribution of contact areas may be described by a power-law
7.5
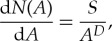

where *N*(*A*) is the total number of contacts of area equal to or smaller than *A*, where *S* and *D* are constants. The fraction of contact area that is wet may then be given by the relationship
7.6
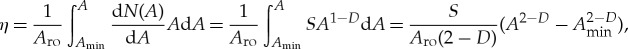

where *A*_ro_ denotes the initial real contact area and *A*_min_ denotes the smallest contact area in the distribution. Square-shaped contacts of area *A* = (2*a*)^2^ are related through equation (7.3) to the corresponding critical velocities through the equality 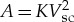, where *K* is a constant and a function of a number of thermal parameters, *K* = *f*(*κρC*_p_Δ*TL*_v_*δ*).

Thus, from equations (7.4) and (7.6), and assuming that meltwater offers insignificant resistance to sliding within the velocity-weakening regime, the coefficient of kinetic friction versus velocity may then be expressed by the relationship
7.7
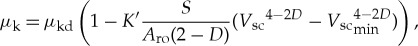

where *K*^′^ is another constant related to *K* and *V*_scmin_ denotes the velocity at which the meltwater remains on the smallest contact.

Equation (7.7) captures velocity weakening (for *D* ≠ 2). But how well does the relationship account quantitatively for the actual kinetic coefficient? We do not know, because at this juncture values of the distribution parameters *S* and *D* are not known. However, if it is assumed that the above depiction-cum-mechanism is close to the truth, then the data plotted in figure 8 can be used to derive a value for *D*. Accordingly, by rewriting equation (7.7) and then taking logarithms
7.8
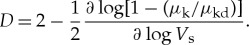

Through separate analysis, and using equation (7.1) to obtain appropriate velocity-dependent values for *μ*_kd_, a double-log plot of the velocity-weakening data in figure 8 yielded the parametric value *D *= 1.90 ± 0.05 for both the smoother and rougher interfaces. While we caution against putting too fine a point on this value, we note that closely similar *D-*values have been obtained for quartz, calcite, glass and acrylic plastic from direct observations of contact areas on prepared and roughened surfaces [74].

Until the distribution parameters *S* and *D* are directly determined, the forgoing model remains untested. Nevertheless, although roughness is again absent from the picture and fracture is not directly incorporated, the fractal-base model offers a new interpretation of velocity weakening.

## 8. Implications for sea-ice mechanics

### (a) Static healing

How much creep-driven healing—i.e. the recovery of strength in the absence of ‘welding’ through the freezing of sea water—is expected within the arctic sea ice cover during periods of dormancy? If one assumes that the underlying physics is scale-independent and that parametric values are similar to those derived in the laboratory, then the analysis given in §5 suggests that only about 5% of the original strength may be recovered upon holding damaged ice at −10°C for a period of 10 days under a low confining stress of 20 kPa, comparable to the stress measured [14] within the winter ice cover. A higher degree of about 50% is expected after the same period under a higher stress of 200 kPa. The presence of sea water, barring the creation of welded joints, is expected to exert little influence on static healing, given that the rate of static strengthening is essentially the same in both wet and dry environments [21]. In other words, whether stress-driven, solid-state healing contributes significantly to the restoration of compressive strength to the winter sea-ice cover depends on the level and the duration of confinement and on the temperature of the ice.

### (b) Stable versus unstable sliding

Returning to figure 1 and to the questions the image raises, it is now clear that the stress to initiate sliding after a dormant period under a non-zero compressive load increases with holding time. Given the intermittent character of sea-ice deformation [13,75,76], the dormant period is expected to vary, perhaps from as little as a few minutes to as long as a few days or more. Correspondingly, given the logarithmic dependence of static strengthening on hold time, the coefficient of static friction is expected to vary by a factor of four or more, assuming that the functionality derived from sliding in the laboratory applies to sliding in the field. Once initiated, sliding is expected to be resisted by a friction force whose magnitude first increases as the velocity picks up and then, once a critical velocity of around 10 m d^−1^ is reached, decreases as the velocity increases further to around 10 km d^−1^. Based upon the response of the unaided ear during experiments in the laboratory [48], increased noise is expected to accompany the increase in sliding speed; but whether noisier sliding correlates with the transition from velocity strengthening to velocity weakening is not known. The idea that velocity-weakening sets in when frictional heating leads to localized melting of asperities leads to the expectation that the critical velocity increases as the temperature of the ice decreases, scaling as Δ*T*^2^. A difference in temperature of say 20°C from the upper/colder layers to the lower/warmer layers of a floating ice sheet is then expected to spread the transition velocity by two to three orders of magnitude, such that under some conditions the upper part of the sheet, of greater Δ*T*, may slide within the strengthening regime while the lower part of smaller Δ*T* may slide within the weakening regime. The temperature difference *per se* is expected also to lead to different values of the coefficient of kinetic friction across the thickness of the sheet. Over the sliding speeds that have been measured within the sea-ice cover—i.e. from almost nothing to up to around 10 km d^−1^—the coefficient of kinetic friction is expected to vary by as much as an order of magnitude. In other words, sliding is expected to be initially relatively quiet and stable, but then to become noisier and unstable. Episodes of sudden slip are thus expected as wind-driven stresses build-up. Superimposed on this stable-to-unstable transition is stick slip where sliding is expected to occur in a jerky manner, possibly on both the macroscopic and microscopic spatial scales. Whether the interface is submerged is expected not to affect significantly the resistance to sliding. Rubble of the kind evident in figure 1, if present at the interface, is expected to impede sliding, but at this juncture the magnitude of the impediment is not known.

### (c) Sliding distance

From the perspective of the arctic sea-ice cover, sliding is expected to occur on all scales, from the sub-millimetre, as related to transgranular cracks and brittle compressive failure, to tens of metres and greater, as related to linear kinematic features/Coulombic faults. There is little to suggest that the coefficient of kinetic friction changes significantly upon sliding a few millimetres. Whether sliding over a much greater distance has an effect is an open question.

## 9. Summary and concluding comment

Based largely upon a critical review of the literature, but including a more quantitative, physics-based analysis of static friction and an analysis of kinetic friction that incorporates the (proposed) fractal character of the sliding interface, the friction of sea ice may be summarized as follows.

1. Following a short threshold period during which no increase is detected, the coefficient of static friction increases with hold time under normal load, scaling with the logarithm of time. The coefficient can be understood in terms of the creep and fracture of asperities in contact, leading to a model that agrees closely with measurement.
2. The coefficient of low-speed (less than or equal to 0.1 m s^−1)^ kinetic friction exhibits velocity strengthening at lower speeds (below approx. 10^−5^ to 10^−4^ m s^−1^) and velocity weakening at higher speeds. Velocity strengthening can be understood in terms of asperity creep and then quantitatively modelled in terms of that process. Velocity weakening can be understood in terms of a progressive increase in the true area of contact that is wetted by meltwater produced through frictional heating. The smallest contacts melt first which leads to the onset of weakening; weakening ends when the largest contacts melt, leading to a third regime of kinetic friction where hydrodynamics governs.
3. Neither the static nor the kinetic coefficient is significantly affected by the presence of sea water.
4. Much of what we understand about the physics of ice/ice friction is based upon supposition. To advance, more direct observations are needed, particularly of the interface.

## Data accessibility

This article has no additional data.
